# Endometriosis During Adolescence: A Narrative Review

**DOI:** 10.3390/jcm14217755

**Published:** 2025-10-31

**Authors:** Caroline Ruth Mathias, George Condous, Mercedes Espada Vaquero

**Affiliations:** Department of Obstetrics and Gynaecology, Nepean Hospital, Kingswood, NSW 2747, Australia; caroline.mathias@health.nsw.gov.au (C.R.M.);

**Keywords:** endometriosis, diagnosis, adolescence, imaging, ultrasound, adenomyosis, dysmenorrhoea

## Abstract

Endometriosis in adolescents presents unique diagnostic and management challenges compared to its manifestation in adult women. The symptoms can vary significantly among individuals, often resulting in a substantial delay in diagnosis, which subsequently impairs quality of life in this vulnerable age group and leads to worsening symptoms and considerable debilitation in adulthood. Recent diagnostic modalities and biomarkers, along with emerging treatment strategies, offer hope for a more evidence-based approach. However, much research is still needed to understand the causality and progression of the condition to aid in the development of targeted treatments. This narrative review aims to provide current insights and clinical considerations for endometriosis in adolescents and discuss unresolved questions that may encourage future research in the field.

## 1. Introduction

Endometriosis is a complex condition characterized by the presence of endometrial glands and stroma outside the uterus, typically within the peritoneal cavity of the pelvis, and occasionally in extra-pelvic sites such as the diaphragm, small bowel, appendix, lungs, and brain [1,2]. While research on endometriosis in adults has progressed significantly over the past two decades, studies on adolescent endometriosis are still in their early stages, with limited evidence available regarding optimal diagnostic and management strategies [3].

Diagnosing endometriosis in adolescents presents challenges, often resulting in the disease being overlooked for several years. Misdiagnosis can significantly delay referral to specialized tertiary centres, potentially leading to disease progression, decreased quality of life, and further fertility impairment. Dysmenorrhea, which may be primary or secondary, is commonly reported among adolescents with endometriosis, making the distinction between the two forms crucial for clinicians [4].

The pathogenesis of deep endometriosis (DE) and its early onset during adolescence remains a subject of debate. Two main theories have been proposed to explain the histological mechanisms involved in the development of endometriosis: one posits that normal cells undergo growth, transformation, or metaplasia due to local or immunologic factors; the other suggests that DE may arise from genetically or epigenetically modified cells [5,6,7].

This narrative review aims to examine the estimated prevalence, unique clinical characteristics, risk factors, progression, and clinical impact of endometriosis in adolescents. Special emphasis is placed on current insights and advancements in the diagnosis and treatment of this complex condition in young patients.

### Search Methodology

An electronic literature search was conducted using databases like MEDLINE, PubMed, EmBase, and Google Scholar to identify English articles on endometriosis in adolescents from inception to April 2025, with a focus on the most relevant articles in the last decade. Various combinations of MeSH terms such as “endometriosis”, “adolescent/s”, “adenomyosis”, “quality of life” and “diagnostic imaging” were used. Original articles, prospective and retrospective observational studies, review articles and their reference lists were reviewed. Articles covering prevalence, risk factors, symptoms, disease burden, progression, severity, diagnosis, and treatment of endometriosis in adolescents were eligible. Two authors (C.R.M and M.E.V) performed the research independently, including a total of 70 articles in this review.

## 2. Epidemiology

Endometriosis affects about 10% of pre-menopausal women [8], but its prevalence in adolescents is unclear. The rate varies based on study criteria and diagnostic methods, such as imaging and surgery. Medical history can aid early diagnosis despite not being definitive [4,9]. Surveys show symptoms often start in adolescence, with 70% reported before age 20 and 40% before age 15 [10]. Millischer et al.’s study found 39.3% of adolescents with severe dysmenorrhea had ovarian endometriomas (OE) or DE, and 17.3% had adenomyosis [11]. This suggests adenomyosis also affects younger women. Endometriotic lesions increase with age, notably from 18 years old [11]. Severe endometriosis phenotypes are common among adolescents with intense dysmenorrhea [11]. Martire et al.’s analysis confirmed similar rates in adolescents undergoing transvaginal ultrasound (TVUS) for endometriosis [12].

In the review by Oliveira, I.J. et al., even though only 30.6% of adolescents suspected to have endometriosis had positive sonographic signs, it is important to consider endometriosis in adolescents with normal ultrasound results, especially for superficial endometriosis (SE) [13]. Accurate diagnosis should be performed by experienced ultrasound examiners [13,14].

In the study conducted by Jannsen, E.B. et al., based on fifteen selected studies, the overall prevalence of visually confirmed endometriosis at all stages was found to be 62% (543/880; range 25–100%) among all adolescent girls undergoing laparoscopic investigation. Specifically, it was 75% (237/314) in girls with chronic pelvic pain (CPP) resistant to treatment, 70% (102/146) in girls with dysmenorrhea, and 49% (204/420) in girls with CPP that is not necessarily resistant to treatment [15].

### Risk Factors

Endometriosis primarily affects young women. Risk factors include prematurity, Müllerian anomalies, family history, low BMI, early menarche (before age 12), early dysmenorrhea, prolonged menstruation, and short menstrual cycles (less than 28 days) [16,17]. Conversely, oral contraceptives, regular exercise, consumption of omega-3 fatty acids, and menarche after 14 years may reduce the risk [17]. The prevalence is lower in Black and Hispanic women compared to Caucasian and Asian women [18]. Genetic predisposition accounts for about 50% of the likelihood of developing endometriosis, with hormonal, environmental, and lifestyle factors also playing a role [17].

## 3. Clinical Presentation

Adolescents with endometriosis often experience severe dysmenorrhea that is resistant to treatments like contraceptive pills, NSAIDs, or anti-spasmodics [19]. Non-cyclical pelvic pain is also common and, when combined with persistent dysmenorrhea despite medication, strongly indicates endometriosis [9,19,20]. Martire et al. highlighted the underestimation of dysmenorrhea in adolescents, emphasizing the need for specific questions related to dysmenorrhoea to suspect endometriosis [12]. Sexually active adolescents report more severe dyspareunia than older women [21]. Early-stage endometriosis may involve metabolically active lesions, leading to more painful symptoms such as seen in adolescents as opposed to larger more fibrotic lesions [9]. Wuest et al. found that women under 24 with endometriosis had higher visual analogue scale scores for dysmenorrhea and non-cyclical pelvic pain compared to those older than 24 [21].

Di Vasta and Dixon S. et al. found that dysmenorrhea is a presenting symptom of adolescent endometriosis in over 90% of patients [4,22]. It starts at menarche in half of these patients, often accompanied by nausea (69.5%) but rarely vomiting (24.6%), and is severe in 63% of cases. Greene et al. identified associations with non-menstrual pain, pre-menstrual spotting, headache, nausea, and bowel symptoms [23]. Tandoi et al. noted that younger age could indicate a more aggressive form of endometriosis [24]. Adolescents are more likely than adults to experience persistent, drug-resistant chronic pelvic pain (CPP), suggesting endometriosis and potentially central sensitization [25,26,27,28].

Education programmes in some countries aim to raise awareness about endometriosis symptoms including severe dysmenorrhea and non-cyclical pelvic pain among adolescents, promoting early diagnosis through social media and e-learning for both patients and caregivers [29,30].

### Severity and Progression

A 2017 systematic review by Yeung et al. found that most adolescents are diagnosed with ASRM Stage I endometriosis, a result confirmed by Oliveira’s 2025 review [13,31]. Brosens et al. suggested endometriosis may be progressive [9], as supported by Stochino-Loi and Millischer who noted an age-related increase in severity, with adolescents (<20 years old) typically having ASRM Stage II disease [11,32]. No advanced cases were detected by laparoscopy or ultrasound by Di Vasta and Martire [4,12], except for some studies in Oliveira’s review indicating selection bias at major referral centres [13,24,33].

Martire’s studies showed a higher prevalence of posterior and lateral compartment (ovarian fossa and pelvic side walls) involvement in adolescents with endometriosis, while anterior compartment (bladder and ureters) involvement was less prevalent. Among younger adolescents (12–16 years old), there was a lower incidence of posterior DE, OE, and adenomyosis compared to older adolescents (17–20 years old) [12].

Red or clear (or non-pigmented) SE lesions are more biologically active than black, puckered lesions, which are associated with old blood and less metabolic activity. Biologically active lesions also tend to exhibit more vascularization and angiogenesis [9,34]. In the study by Davis G. et al., adolescents were found to have significantly more red lesions than adult women (age group 31–46 years) [34]. The red and clear lesions usually do not have sclerosis or fibrosis in the surrounding areas as found in older, fibrotic lesions in adult patients. Generally, there is no correlation between disease stage and the amount of pain experienced by patients. This is likely determined by the interaction between endometrial lesions and sensory afferent nerve fibres rather than solely the type and extent of the lesions [35].

In summary, all stages of endometriosis can be found in adolescents, with a predominance of ASRM disease stage I/II. This questions the hypothesis that endometriosis in adolescent patients initially consists of ASRM I/II disease, which later progresses to ASRM Stage III/IV disease, varying widely between individuals [17]. Recent data from genome-wide association studies showing different genetic loci for early and advanced stage endometriosis may support the hypothesis that these stages are different clinical entities [36].

The natural history of endometriosis is poorly understood because few patients progress through their reproductive lifespan without attempts at effective therapy [37]. Literature on progression in adolescents is scarce, particularly regarding changes from adolescence to adulthood. Early diagnosis and treatment may limit disease progression, infertility, and central sensitization, but this requires verification through prospective research [38,39].

## 4. Diagnosis

Adolescents with endometriosis experience a significant negative impact on their physical and mental health-related quality of life compared to controls without the condition (Gallagher et al.) [40]. Diagnosis delays can average 8 years, with symptoms in adolescence often extending the delay further, with 65% of women initially misdiagnosed [41]. If symptoms begin in adolescence, this delay has been reported to be up to three times longer [23]. The diagnostic process in adolescents is complex for several reasons. History taking may reveal symptom overlap with normal menstruation [23,42] and physical examination and imaging may fail to detect elusive small foci of SE which forms the main burden of disease in this age group [25]. Adolescents may be encouraged to normalize their symptoms through stigma by their peers, family, or school personnel [43]. The awareness of endometriosis in adolescents in medical professionals and caregivers of adolescents is also low with a lack of insight into its presentation and a hesitance to diagnose adolescents with the disease, further accounting for a delayed diagnosis [20,43]. In this cross-sectional self-reported survey-based study by Greene et al., 63% (n = 4334) of respondents reported being told nothing was wrong by at least one physician at some point while seeking a diagnosis and more than half of the respondents reported that they were not taken seriously by their physicians overall [23]. Ballard et al.’s study highlights factors such as symptom overlap with benign conditions, cultural beliefs, and concerns over laparoscopy that contribute to delayed diagnosis.

To mitigate the delay in diagnosing endometriosis in adolescence and prevent a significant negative impact on their quality of life, healthcare professionals at both primary and secondary levels of care should be thoroughly informed about this condition. If left undiagnosed, endometriosis can result in central sensitization, scarring, CPP, and reduced fecundity [4,11].

### The Paradigm Shift in Diagnosis

Over the past decade, there has been a notable paradigm shift in the diagnosis of endometriosis [39]. There is a growing advocacy for modern, non-invasive diagnostic methods for endometriosis in adults, which combine patient interviews, clinical examinations, and imaging techniques such as transvaginal ultrasonography (TVUS), Trans-rectal ultrasonography (TRUS) and magnetic resonance imaging (MRI). This approach may also be applied to diagnosing endometriosis in adolescents [19].

In a cross-sectional study by Chapron, C. et al., markers for endometriosis in adolescents include a family history of endometriosis, primary severe dysmenorrhea, school absenteeism during menstruation, dysmenorrhea resistant to nonsteroidal anti-inflammatory drugs, and the prescription of oral contraceptives for severe dysmenorrhea [19]. From a clinical examination standpoint, Bryan, A.F. and J. Chor emphasize the importance of considering the utility of pelvic examinations carefully, recognizing that it may be the patient’s first experience with such an examination, which could be perceived negatively by many adolescents and young adults [44].

For suspected endometriosis in adolescents, transabdominal ultrasound (TAUS) is recommended first [45]. It is always feasible, non-invasive, and can exclude other causes of pelvic pain, such as Müllerian anomalies. However, it has limitations in detecting endometriotic lesions, which are better identified via TVUS and TRUS [7]. TAUS may detect OE, a less common form of endometriosis in adolescents [45,46]. Millischer A.E. et al. reported a notably high 40% incidence of OE detected on imaging among adolescents; however, this was derived from a single tertiary-centre cohort (n = 345) and may not accurately represent the true population incidence [11].

TVUS/TRUS is preferred for diagnosing endometriosis, with standardized methods to describe its appearance [14]. It is inexpensive, widely available, and accepted by patients and caregivers [7]. However, adolescents who are not sexually active may find TVUS uncomfortable [13]. In such circumstances, a TRUS approach may be considered and with expertise is similar to TVUS in the imaging and diagnostic accuracy of endometriotic lesions of the pelvis [47]. TRUS has shown good diagnostic performance especially with posterior compartment disease in adults but limited research has been conducted in adolescents [48]. In a study of adolescents using pelvic ultrasound (TVUS if sexually active, TRUS if not), 13.3% showed features of endometriosis, including OE, adenomyosis, and DE [12]. Compared with TVUS, TRUS provides equivalent high accuracy in diagnosing rectosigmoid DE and with an added advantage in precisely determining lesion proximity to the anal verge, aiding in detailed surgical planning [49].

Though transvaginal hydrolaparoscopy (THL) has been suggested, it is not widely used or validated [9]. THL involves the introduction of a needle through the vaginal fornix into the pouch of Douglas, followed by insertion of a trocar and cannula through which a rigid scope is introduced, with the pelvis distended by warm saline solution. This technique allows for the endoscopic evaluation of the pelvic cavity, facilitating the diagnosis of conditions such as endometriosis and adhesions.

Given the limited value of TAUS and the invasive nature of TVUS and TRUS, MRI is useful for symptomatic adolescents [50].

MRI is effective at identifying DE lesions, especially in the posterior compartment like the pouch of Douglas and uterosacral ligaments. It allows multi-plane assessment and retrospective image review for thorough preoperative evaluation [13,50]. A study found retro cervical lesions in 4.7% of patients under 15 years, 30% of patients aged 15–18 years old, and up to 65% of those aged 18–20 years old. No rectosigmoid, bladder, or ureteric lesions were seen in patients younger than 15 years old. OE were observed in 20.7% of patients [11].

However, MRI is less accessible, expensive, and lacks real-time dynamic observation. Its claustrophobic nature may also be less acceptable to anxious adolescents. Currently, TVUS and TRUS offer more acceptable diagnostic options with real-time dynamic observation, revealing adhesions and Pouch of Douglas obliteration [13]. Recent research has highlighted TVUS enhanced role in detecting SE lesions in adults [51].

Overall, both MRI and ultrasound are crucial for diagnosing endometriosis, complementing each other with distinct advantages and limitations [7]. TVUS and MRI are now recognized as suitable for adenomyosis evaluation [52]. They should be the first step in investigating symptomatic adolescents, contrary to previous teachings favouring diagnostic laparoscopy purely for diagnosis. Laparoscopy remains relevant, weighing risks against benefits when imaging is negative and symptoms persist despite medical treatment trials [20].

New strategies, like using micro-RNA and long non-coding RNA as diagnostic biomarkers, are promising [53]. Bendifallah et al. discussed a saliva-based diagnostic miRNA signature for endometriosis [54] and the ENDOmiRNA saliva test study’s interim analysis paper has shown promising results with a sensitivity of 96.2% (95% confidence interval [CI], 93.7 to 97.3%), specificity of 95.1% (95% CI, 85.2 to 99.1%), positive predictive value of 95.1% (95% CI, 85.2 to 99.1%) and a negative predictive value of 86.7% (95% CI, 77.6 to 90.3%) [55]. Saliva sampling is cheap, acceptable, non-invasive, and can be repeated, potentially improving diagnostic and therapeutic management through early identification [54]. However, the high cost of the test may pose a significant barrier for patients. Sasamoto et al. identified circulating proteins and biological pathways linked to laparoscopically confirmed endometriosis in adolescents and young adults, revealing molecular characteristics that could guide future diagnostic studies [56].

## 5. Treatment

**Figure 1:**
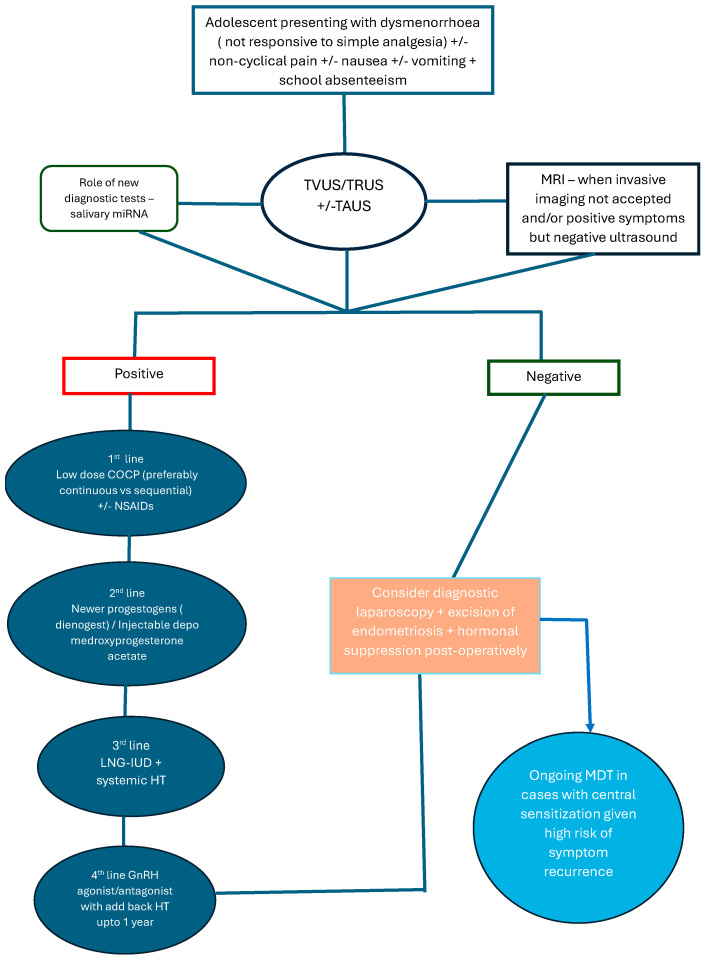
Flowchart for the management of endometriosis in adolescents. Abbreviations: TVUS—trans-vaginal ultrasound, TRUS—trans-rectal ultrasound, TAUS—transabdominal ultrasound, MRI—magnetic resonance imaging, miRNA—microRNA, COCP—combined oral contraceptive pill, GnRH—gonadotropin releasing hormone, LNG-IUD—levonorgestrel intrauterine device, HT—hormonal therapy, MDT—multi-disciplinary team.

The primary objectives of treating endometriosis in adolescents are to alleviate painful symptoms, control disease progression, avoid symptom recurrence and protect future fertility [16] (Figure 1).

High-quality evidence on the efficacy of medical treatment for endometriosis in adolescents is currently scarce [20]. Non-steroidal anti-inflammatory drugs (NSAIDs) have proven effective in managing symptoms; however, clinical evidence supporting their use specifically in adolescents is limited. Nonetheless, they may serve as a first-line treatment in cases where hormonal management is intolerable [20].

### 5.1. Medical Management

Combined oral contraceptive pills (COCP) and/or progestins should be the initial choice for long-term medical management of endometriosis in adolescents [20,45]. COCP are recommended as first-line medical therapy for adolescents with endometriosis because they suppress ovulation and reduce menstrual-related pain; continuous regimens are preferred over cyclical/sequential use to provide more consistent endometrial suppression and minimize withdrawal bleeding and breakthrough symptoms [42]. The selection of treatment should consider various factors, including contraceptive needs, daily activities and schedule, contraindications or preferences to avoid estrogen-containing therapy, and the patient’s willingness and ability to tolerate the method, such as swallowing pills or device insertion [45].

In a randomized controlled trial by Davis et al., a low-dose COCP containing ethinylestradiol 20 mcg/levonorgestrel 100 mcg was more effective than a placebo in controlling dysmenorrhea-associated pain [57]. In the VISADO study conducted by Ebert, A.D. et al., adolescents with suspected endometriosis were treated with dienogest 2 mg for 52 weeks [58]. This treatment significantly reduced endometriosis-associated pain but was associated with a decrease in lumbar bone mineral density (BMD), followed by partial recovery after discontinuation. Despite the efficacy, these findings underscore the need for tailored treatment approaches. Similarly, injectable depot medroxyprogesterone acetate has been shown to effectively reduce dysmenorrhea and pelvic pain in adolescents with endometriosis, but long-term use may be associated with decreased BMD, necessitating monitoring during therapy [59].

Regarding the use of the levonorgestrel intra-uterine device (LNG-IUD), a study by Shim, J.Y. et al., involving adolescents with laparoscopically confirmed endometriosis reported that most participants used the LNG-IUD alongside additional systemic hormonal contraceptive therapy (HCT). Those who combined LNG-IUD with HCT were more likely to continue LNG-IUD use compared to those not utilizing additional HCT [60].

In adolescents with laparoscopically confirmed endometriosis and associated pain where COCP or progestogen therapy are ineffective for symptom control, clinicians may consider prescribing GnRH agonists for up to 1 year. These are effective and safe when combined with add-back therapy [61]. In a study by Sadler Gallagher et al., female adolescents treated with GnRH agonists combined with add-back therapy showed improved quality of life, with no worsening of mood or menopausal side effects. Add-back therapy with norethindrone acetate and conjugated equine estrogen was found to be superior for improving physical health-related quality of life compared to norethindrone acetate alone [61].

The use of GnRH receptor antagonists combined with add-back therapy in the SPIRIT 1 and 2 trials has demonstrated significant efficacy and safety in managing endometriosis symptoms in adult women, though this remains to be established in the adolescent age group [62].

In conclusion, there is no single best medical treatment for endometriosis in adolescents, and therapy must be tailored to each individual.

### 5.2. Surgical Treatment

Optimal timing for surgical treatment of endometriosis in adolescents has yet to be established. Since endometriosis may be a progressive disease [9,20], the decision between treating it early with surgery or using hormonal medical therapy to inactivate disease foci and alleviate pain remains open to discussion [9,12]. Surgery may be required if medical treatment fails, considering associated risks, postoperative complications, and high risk for recurrence [12,17,20].

Scientific evidence is minimal regarding the optimal technique for endometriosis surgery in adolescents. No difference has been demonstrated between excision and ablation for patients with low-stage SE [45]. The review by Shim, J.Y. et al. advises against peritoneal stripping, especially with visually normal peritoneum, given the lack of evidence and high risk of adhesion formation as demonstrated in the case study by Laufer et al. which showed worsening of pelvic pain symptoms after extensive peritoneal excision for SE and adhesion formation [63]. Cystectomy is preferred over simple drainage and excessive use of cautery/CO_2_ laser to surgically manage OE [45,64].

In a study by Dun, E.C. et al., 80% of adolescents reported resolved or improved pain outcomes at 1-year follow-up after laparoscopic treatment of ASRM stages I–III endometriosis with postoperative hormonal suppression [3]. Another study by Doyle, J.O. et al. showed no observation of disease progression at subsequent laparoscopy after 2–10 years, with 70% showing no stage change and most patients with initial stage 2 and 3 disease showing improvement [65]. However, the reason for subsequent laparoscopy in this study was due to symptom recurrence. This compares to studies by J.D. Roman and Yeung, P. et al. on improved symptomology post-surgery but recurrent symptoms over time with minimal or no evidence of recurrent lesions [66,67]. Hence, anticipatory counselling of adolescent patients with endometriosis regarding this aspect remains essential. Addressing alternate pelvic pain generators and including a multidisciplinary team before and after surgery can optimize outcomes [68], given the likelihood of central sensitization and prefrontal cortex remodelling that may explain ongoing pain despite surgical excision [69].

A retrospective cohort study with a sample size of 176 adolescents by Seo, J.Y. et al. showed long-term postoperative medical treatment with cyclic COCP after a GnRH agonist to be as effective in adolescents as in adults in preventing OE recurrence [70]. Smaller studies have shown lesion recurrence at subsequent laparoscopy in adolescents after longer follow-up, with no conclusions drawn about risk profiles in this patient subset. No association was noted between recurrence rate and endometriosis-related symptoms, site/stage of the disease, type of surgery, and post-surgical medical treatment [24,33].

In conclusion, clinicians are recommended to consider post-surgical hormonal suppression to prevent lesion and symptom recurrence for a period of 18–24 months that has proven to be longer than duration until recurrence without hormonal therapy [20]. Currently, no established relevance or efficacy exists for oocyte preservation in adolescents with endometriosis (especially OE), but adequate counselling on potential harm from OE and subsequent surgery on future fertility is recommended [20].

## 6. Conclusions

Endometriosis in adolescents remains an under-researched and challenging condition. Timely diagnosis is crucial and can be achieved through a combination of medical history review, physical examination, and modern imaging techniques. Early referral to a tertiary care centre and a multidisciplinary approach is advised for optimal management of this complex, debilitating, and chronic disease. Patients and their families should receive quality education and comprehensive information regarding all management options.

## 7. Future Directions

Additional research is essential for understanding disease progression, determining the optimal timing for surgical intervention, assessing the effects of hormonal suppression, identifying genetic factors associated with the disease, and utilizing biomarkers in diagnosis. Artificial intelligence (AI) applications are being explored to improve the early recognition and management of endometriosis in adolescents, including machine learning analysis of ultrasound and MRI images (e.g., IMAGENDO^®^, EndoKI, Matricis.ai) and AI-driven symptom self-assessment tools (e.g., Helfie AI, ANNA project). Although many of these tools are still in development or undergoing validation, they hold promise for reducing diagnostic delays, supporting personalized management, and enhancing patient education. Furthermore, establishing distinct risk profiles for adolescent patients with endometriosis will be critical to predict disease progression, symptoms and lesion recurrence, the likelihood of central sensitization and potential subfertility risk.This narrative review provides a thorough understanding of endometriosis in adolescents, offering insights into disease prevalence, clinical burden, symptomatology, severity and progression, diagnostic modalities, new innovations, treatment strategies, and existing knowledge gaps that necessitate further research.

## Data Availability

Not applicable.
